# Point-of-Injury Treatment with Hydrogel Containing Dexamethasone Improves Cognitive Function and Reduces Secondary Injury Response After TBI

**DOI:** 10.3390/gels11080600

**Published:** 2025-08-01

**Authors:** Claire E. Jones, Bradley Elliott, Fuying Ma, Zachary Bailey, Janice Gilsdorf, Anke H. Scultetus, Deborah Shear, Ken Webb, Jeoung Soo Lee

**Affiliations:** 1 Drug Design, Development and Delivery Laboratory, Department of Bioengineering, Clemson University, Clemson, SC 29634, USA; claire.e.jonesresearch@gmail.com (C.E.J.); bmellio@g.clemson.edu (B.E.);; 2 Brain Trauma Neuroprotection Branch, Walter Reed Army Institute of Research (WRAIR), Silver Spring, MD 20910, USAjanice.s.gilsdorf.civ@health.mil (J.G.); anke.h.scultetus2.civ@health.mil (A.H.S.);; 3 MicroEnvironmental Engineering Laboratory, Department of Bioengineering, Clemson University, Clemson, SC 29634, USA; kwebb@clemson.edu

**Keywords:** traumatic brain injury (TBI), PEG-bis-AA/HA-DXM hydrogel, dexamethasone, secondary injury, cognitive function recovery

## Abstract

Functional recovery after traumatic brain injury (TBI) is hindered by progressive neurodegeneration resulting from neuroinflammation and other secondary injury processes. Dexamethasone (DX), a synthetic glucocorticoid, has been shown to reduce inflammation, but its systemic administration can cause a myriad of other medical issues. We aim to provide a local, sustained treatment of DX for TBI. Previously, we demonstrated that PEG-bis-AA/HA-DXM hydrogels composed of polyethyleneglycol-bis-(acryloyloxy acetate) (PEG-bis-AA) and dexamethasone-conjugated hyaluronic acid (HA-DXM) reduced secondary injury and improved motor functional recovery at 7 days post-injury (DPI) in a rat moderate controlled cortical impact (CCI) TBI model. In this study, we evaluated the effect of PEG-bis-AA/HA-DXM hydrogel on cognitive function and secondary injury at 14 DPI. Immediately after injury, hydrogel disks were placed on the surface of the injured cortex. Cognitive function was evaluated using the Morris Water Maze test, and secondary injury was evaluated by histological analysis. The hydrogel treatment group demonstrated significantly shorter latency to target, decreased distance to find the hidden target, increased number of target crossings, increased number of entries to the platform zone, and decreased latency to first entry of target zone compared to untreated TBI rats for probe test. We also observed reduced lesion volume, inflammatory response, and apoptosis in the hydrogel treatment group compared to the untreated TBI group.

## 1. Introduction

Traumatic brain injury (TBI) damages neural tissue through at least two distinct injury stages. The initial trauma results in mechanical damage to brain tissue, particularly shear force that damages cell membranes and axonal projections. This is followed by a prolonged and progressive secondary injury process characterized by excitotoxicity, neuroinflammation, and oxidative stress that leads to continued damage to the tissue [1]. The progression of the secondary injury manifests as altered motor, sensory, or cognitive skills, or psychological changes [2,3,4,5]. Retrospective long-term studies show that TBI increases mortality risk and incidence of mental health disorders and neurodegenerative disease, with key risk factors being injury severity and patient age [6]. Lifelong costs associated with TBI total approximately USD 76.5 billion in direct and indirect expenses [7]. Moderate to severe TBIs result in hospitalization where patients are monitored for intracranial pressure, blood flow, and excitotoxicity. Surgical interventions include removing blood clots, decompressive craniectomy, implantation of a monitoring device and/or implantation of ventriculoperitoneal shunts to relieve pressure [8]. Currently, there is no established drug therapy targeting the underlying pathophysiology of TBI, but symptoms are managed through the use of steroids, antianxiety medications, anticoagulants, anticonvulsants, antidepressants, diuretics, muscle relaxants, or stimulants [9,10].

Current preclinical research is focused on discovering and developing therapeutic treatments that can diminish the inflammatory reaction and reduce its deleterious effects on the neural microenvironment [10,11]. Secondary injury is characterized by increased blood–brain barrier (BBB) permeability, infiltration and activation of monocytes/macrophages, glial cell activation, inflammatory cytokine expression, and apoptosis [12,13,14]. Activated astrocytes upregulate the expression of glial fibrillar acidic protein (GFAP), an intermediate filament protein, and form a barrier that limits neurotrauma and preserves neuronal synapses by reducing the spread of inflammatory cytokines, reducing excitotoxicity, and supporting recovery of the extracellular matrix [14,15,16,17,18,19,20]. These reactive astrocytes also contribute to the formation of a glial scar that can limit recovery through reduced neuroplasticity, inhibition of axonal regeneration through release of inhibitory growth molecules such as chondroitin and keratan sulfate proteoglycans, and promotion of cytotoxic edema [14,16,21,22,23]. By 10–14 days post-injury (DPI), there is a decrease in the number of infiltrated macrophages and reactive astrocytes at the site of injury, but the detrimental results from the glial scar can be observed [12,24,25,26]. It is notable that multiple studies have shown that blocking macrophage entry and eliminating astrocyte activation completely after injury did not improve neurological outcomes [12,20,27]. This leads to the potential for anti-inflammatory therapeutics in the acute phase to limit the activation of glial cells and modulate the molecular components of injury-induced inflammation [28,29].

Dexamethasone (DX), a synthetic glucocorticoid steroid, has previously been investigated to limit the neuroinflammatory response after TBI [30,31]. Several studies indicate the potential to decrease pro-inflammatory cytokine expression, reduce neuronal damage, and decrease activation of astroglia [32,33]. Unfortunately, systemic administration of high doses required for therapeutic effect negatively impacts multiple organs and organ systems [34]. Furthermore, when used to treat TBI patients clinically, there was an observed significant increase in risk of infection, chance of disability, and mortality rate [35,36,37].

To overcome the severe side effects associated with high dose systemic administration of DX, we developed an approach for local, sustained DX delivery consisting of a hydrolytically degradable polyethylene glycol-bis-acryloyloxy acetate (PEG-bis-AA) hydrogel loaded with DX-conjugated high molecular weight hyaluronic acid (HA-DXM) [38,39]. In previous work, we reported that PEG-bis-AA/HA-DXM hydrogel treatment reduced secondary injury and improved motor and cognitive function in a rat mild controlled cortical impact (CCI) TBI model [40,41]. We also reported that PEG-bis-AA/HA-DXM hydrogel treatment can improve motor function and inhibit secondary injury in a rat moderate-CCI TBI model at 7 DPI (acute injury phase) [42]. In this study, we evaluated the effect of PEG-bis-AA/HA-DXM hydrogel treatment on cognitive function recovery using Morris Water Maze (MWM) test and secondary injury by histological analysis at 14 DPI (chronic injury phase) in a rat moderate-CCI TBI model.

## 2. Results

### 2.1. PEG-Bis-AA/HA-DXM Improves Cognitive Function

**Figure 1:**
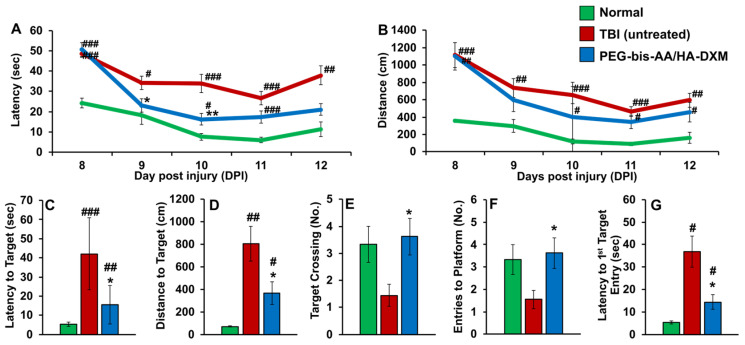
Effect of PEG-bis-AA/HA-DXM on cognitive function by MWM test. (**A**,**B**). Training period (Days 8–12): latency to target (**A**) and distance to target (**B**). (**C**–**G**). Probe test (Day 14): latency to target (**C**), distance to target (**D**), number of target crossings (**E**), number of entries (**F**), and latency to 1st entry (**G**). Data are presented as mean +/− SEM. Normal group (green, n = 3), TBI untreated group (red, n = 9), and PEG-bis-AA/HA-DXM gel-treated group (blue, n = 8); ^#^ *p* < 0.05; ^##^ *p* <0.01; ^###^ *p* < 0.001 compared to Normal, * *p* < 0.05; ** *p* < 0.01 compared to TBI.

The effect of PEG-bis-AA/HA-DXM on cognitive function recovery was evaluated by MWM test. During the training days, PEG-bis-AA/HA-DXM-treated rats showed decreased time (Figure 1A) and decreased distance to find hidden target (Figure 1B) compared to the untreated TBI group. For the probe test at 14 DPI, PEG-bis-AA/HA-DXM-treated rats showed significantly shorter latency to target (Figure 1C), decreased distance to find the hidden target (Figure 1D), increased number of target crossings (Figure 1E), increased number of entries to the platform zone (Figure 1F), and decreased latency to first entry of target zone (Figure 1G) compared to untreated TBI rats.

### 2.2. PEG-Bis-AA/HA-DXM Reduces Lesion Volume

**Figure 2:**
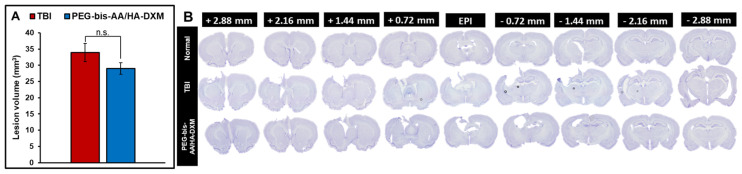
Effect of PEG-bis-AA/HA-DXM on lesion volume by Nissl stain. (**A**) Average lesion volume calculated by Cavalieri’s approximation (n = 19 Sections/rat). Data are presented as mean +/− SEM. TBI untreated group (n = 9), and PEG-bis-AA/HA-DXM hydrogel-treated group (n = 8). (**B**) Representative images of Nissl-stained sections from each group. n.s.: no statistical difference.

The effect of PEG-bis-AA/HA-DXM hydrogel treatment on lesion volume was determined using Cavalier’s formula of approximation based on area of lesion in serial sections. PEG-bis-AA/HA-DXM hydrogel treatment groups showed slightly decreased lesion volume compared to that in TBI untreated group, even though it was not significantly different (Figure 2).

### 2.3. PEG-Bis-AA/HA-DXM Improves Neuronal Cell Survival

**Figure 3:**
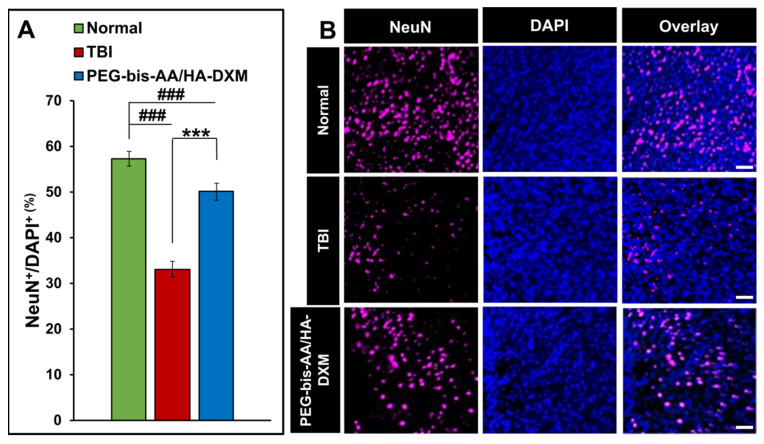
Effect of PEG-bis-AA/HA-DXM hydrogel on neuronal survival by NeuN staining. (**A**) % NeuN^+^ cells normalized to number of total cells (DAPI^+^). Data are presented as mean +/− SEM. Normal group (n = 3), TBI untreated group (n = 9), and PEG-bis-AA/HA-DXM gel-treated group (n = 8); ^###^ *p* < 0.001 compared to Normal; *** *p* < 0.001 compared to TBI. (**B**) Representative images of NeuN^+^ cells (red) in various groups with DAPI nuclear counter-staining (blue). Scale bar = 500 μm.

The effect of PEG-bis-AA/HA-DXM on neuronal cell survival was evaluated by IHC for NeuN. The percentage of NeuN^+^ cells in the PEG-bis-AA/HA-DXM-treated group was significantly increased compared to that in the untreated TBI group (*p* < 0.001) (Figure 3A). Figure 3B shows representative images of NeuN^+^ cells in various groups with DAPI nuclear counter-staining.

### 2.4. PEG-Bis-AA/HA-DXM Reduces Astrogliosis

**Figure 4:**
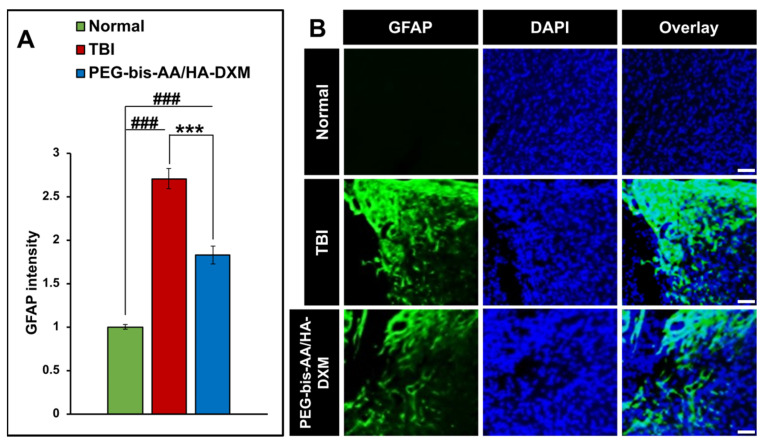
Effect of PEG-bis-AA/HA-DXM hydrogel on astrogliosis. (**A**) GFAP staining intensity normalized to area (mm^2^). Data are presented as mean +/− SEM. Normal group (n = 3), TBI untreated group (n = 9), and PEG-bis-AA/HA-DXM gel-treated group (n = 8); ^###^ *p* < 0.001 compared to Normal; *** *p* < 0.001 compared to TBI. (**B**) Representative images of GFAP staining (green) and with DAPI nuclear stain (blue). Scale bar = 500 μm.

The effect of PEG-bis-AA/HA-DXM hydrogel treatment on astrogliosis was evaluated by measuring the intensity of GFAP immunohistochemical staining in sections. We observed that the PEG-bis-AA/HA-DXM hydrogel-treated group showed significantly lower GFAP staining intensity compared to the untreated TBI group (Figure 4).

### 2.5. Effect of PEG-Bis-AA/HA-DXM on the Inflammatory Response

**Figure 5:**
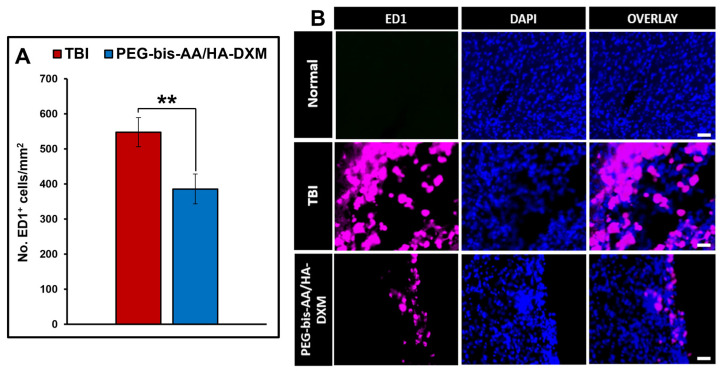
Effect of PEG-bis-AA/HA-DXM on the inflammatory response. (**A**) Number of ED1^+^ cells normalized to area (mm^2^). Data are presented as mean +/− SEM. TBI untreated group (n = 9), and PEG-bis-AA/HA-DXM gel-treated group (n = 8); ** *p* < 0.01 compared to TBI. (**B**) representative images of ED1^+^ cells (red) with DAPI (blue) counter-staining. Scale bar = 500 μm.

The effect of PEG-bis-AA/HA-DXM on the inflammatory response was evaluated by IHC using the ED1 antibody to identify infiltrated peripheral macrophages and activated microglia. The number of ED1^+^ cells in the PEG-bis-AA/HA-DXM hydrogel-treated group was significantly reduced relative to the untreated TBI group (*p* < 0.01) (Figure 5A). Figure 5B shows representative images of ED1^+^ cells in various groups.

### 2.6. Effect of PEG-Bis-AA/HA-DXM on Apoptosis

**Figure 6:**
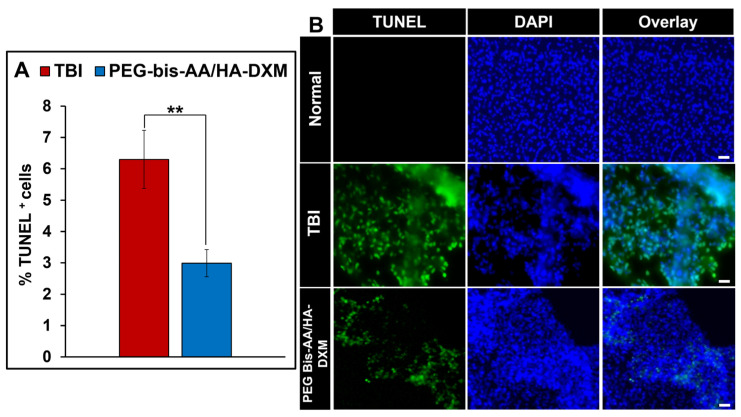
Effect of PEG-bis-AA/HA-DXM on apoptosis. (**A**) % TUNEL^+^ cells. Data are presented as mean +/− SEM. TBI untreated group (n = 9) and PEG-bis-AA/HA-DXM gel-treated group (n = 8); ** *p* < 0.01 compared to TBI. (**B**) Representative image of TUNEL^+^ cells (green) with DAPI (blue) counter-staining. Scale bar = 500 μm.

The effect of PEG-bis-AA/HA-DXM on apoptosis was evaluated by TUNEL staining. The percentage of TUNEL^+^ cells in PEG-bis-AA/HA-DXM hydrogel treatment group was significantly decreased compared to that in untreated TBI group (*p* < 0.01) (Figure 6A). Figure 6B shows representative images of TUNEL^+^ cells in various groups.

## 3. Discussion

TBI exhibits a progressive pathophysiology in which the initial mechanical injury is followed by a complex secondary injury process involving edema, inflammation, astrogliosis, oxidative stress, and excitotoxicity. A major cause of neuronal cell death after TBI is neuroinflammation, and many preclinical studies focus on minimizing damage resulting from the inflammatory reaction [10,23,38]. Glucocorticoids, including dexamethasone (DX), are potent anti-inflammatory drugs, and DX has shown promising results for reducing pro-inflammatory cytokine expression and histological markers of neurotrauma, as well as strengthening the BBB through interaction with tight junction proteins, adherens junctions, and tissue inhibitors of metalloproteinases in preclinical studies [32,33,39,43,44,45,46]. Conversely, high systemic doses of DX showed a high mortality rate and severe deleterious side effects, which has effectively decreased enthusiasm for steroid treatment in TBI clinical studies [35]. In addition, preclinical studies using high dose DX have reported adverse impacts on cognitive function compared to TBI (untreated) [47,48] Zhang et al. treated CCI TBI rats with high dose DX for 7 consecutive days (10 mg/kg for the first three days, 5 mg/kg on day 4, 2 mg/kg on days 5–6, and 1 mg/kg on day 7) and reported that it aggravated the neurological and spatial learning impairment induced by TBI [49]. Chen et al. reported that in TBI rats, high-dose DX (0.5–10 mg/kg) increased neuronal apoptosis in the hippocampus and aggravated retrograde memory deficits induced by TBI [50].

In our previously published studies, we demonstrated that local and sustained delivery of low-dose DX (3 μg/rat) by PEG-bis-AA/HA-DXM hydrogel can improve motor and cognitive function and reduce secondary injury in a rat mild-CCI TBI model [40,41]. We also demonstrated that local and sustained delivery of DX by PEG-bis-AA/HA-DXM hydrogel can improve motor function and reduce secondary injury at 7 DPI (acute phase injury) in a rat moderate-CCI TBI model [42]. In this study, we evaluated the therapeutic efficacy of PEG-bis-AA/HA-DXM hydrogel by evaluating cognitive function and secondary injury at 14 DPI (chronic injury phase) in a rat moderate-CCI TBI model. Treatment with PEG-bis-AA/HA-DXM hydrogel significantly improved spatial learning and memory following TBI, as evidenced by reduced latency and distance to find the hidden platform during training on 9–12 DPI, with pronounced effects on the final day. In the probe test, treated rats demonstrated enhanced memory retention, with decreased latency and distance to target and increased platform crossings compared to TBI untreated group, highlighting the therapeutic potential of the hydrogel for cognitive recovery after TBI. Increased visuospatial pattern recognition decreases latency to target and distance to target indicating a potential increase in recovery in the treatment group. These results are similar to those using this hydrogel treatment in a mild-CCI model and suggest that local, low-dose DX treatment by hydrogel may have therapeutic potential in mitigating the adverse impact of TBI on cognitive function [41].

After cognitive function testing at 14 DPI, we investigated the effect of PEG-bis-AA/HA-DXM on secondary injury by histological analysis. We first observed that lesion volume was decreased in the hydrogel-treated group compared to that in the untreated TBI group, even though it was not significantly different. In our previous study, we observed a significant decrease in the lesion volume at 7 DPI in moderate-CCI TBI model [42]. In our other previous study in a rat mild injury model, we also observed a significant decrease in lesion volume at 7 DPI, but a non-significant decrease at 14 DPI [40,41]. One likely explanation is that the PEG-bis-AA hydrogel composition at 6% *w*/*v* used in these studies is largely degraded and DX fully released within 7 days [40,41]. This suggests that a modified gel formulation or a design able to sustain DX delivery for a longer time period may be beneficial to further improve outcomes in the chronic injury phase.

For neuronal survival, we observed that hydrogel-treated groups showed a significantly higher percentage of NeuN^+^ cells compared to that in the untreated TBI group. This suggests a neuroprotective characteristic of PEG-bis-AA/HA-DXM hydrogel, and this result is consistent with our previous study in a mild-CCI TBI model for neuroprotective effect of PEG-bis-AA/HA-DXM hydrogel [40,41].

Increased astrocyte activation after injury can lead to glial scar formation that reduces neuroplasticity and function [25,51]. Although gliosis serves beneficial roles in the acute phase, such as limiting tissue damage and supporting repair, excessive or prolonged astrocytic activation may hinder long-term regenerative processes [18,19,20,52]. In moderate TBI, astrocyte activity begins to resolve at the 14 DPI time point [25]. The PEG-bis-AA/HA-DXM hydrogel-treated group showed a significantly reduced fluorescent intensity of GFAP^+^ cells compared to that in the untreated TBI group. This result was consistent with our previous observations at 7 DPI in the moderate-injury model [42]. We think that reduction in astrocyte activity in the early days could lead to greater synaptic plasticity in the area that could support improved recovery or ability to recover functional activity in the tissue.

We also observed that PEG-bis-AA/HA-DXM hydrogel treatment reduced the number of ED1^+^ cells. This result is consistent with our previous study in a mild-CCI injury model in which there was a significant decrease in ED1^+^ cells at 14 DPI [41]. Infiltrating peripheral macrophages, as well as neutrophils, are an important source of pro-inflammatory cytokines that are key drivers of neuroinflammation and tissue damage. We believe that local sustained delivery of low-dose DX by hydrogel can reduce the number of infiltrating macrophages in the lesion site and can be potential therapy for TBI.

Apoptosis is one of the key features of secondary injury and continues due to prolonged neuroinflammation [53,54,55]. We observed a significant decrease in TUNEL^+^ cells in the PEG-bis-AA/HA-DXM treatment group compared to that in the untreated TBI group. Additionally, this significant decrease was also observed in our previously published studies in which there is a significant decrease in TUNEL^+^ cells at 7 DPI time point [42] in the moderate injury model as well as at 7 and 14 DPI in the mild injury [40,41]. This suggests that local, hydrogel-mediated DX delivery has a strong neuroprotective effect against the apoptotic pathway after TBI.

In summary, PEG-bis-AA/HA-DXM hydrogel treatment improved cognitive function recovery and many measures of secondary injury at 14 DPI in a moderate TBI model. Table 1 summarizes and compares the findings of the current study with our previous studies of PEG-bis-AA/HA-DXM in mild (7 and 14 DPI) and moderate (7 DPI) TBI models.

## 4. Conclusions

Glucocorticoids in general, and DX in particular, have a long history as promising anti-inflammatory therapeutics for TBI, yet their clinical application has been hindered by challenges associated with high-dose systemic administration. The current study extends our previous work, demonstrating continued efficacy for PEG-bis-AA/HA-DXM in a moderate-TBI model at 14 DPI through improved cognitive function and the mitigation of a wide range of secondary injury responses. These results continue to support the therapeutic potential of DX for TBI when delivered in a local, sustained manner by hydrogels.

## 5. Materials and Methods

### 5.1. PEG-Bis-AA/HA-DXM Hydrogel Preparation

PEG-bis-AA and HA-DXM (containing ~3 mg DX/100 mg HA-DXM) were synthesized and characterized as previously described [47,48]. PEG-bis-AA/HA-DXM hydrogels were prepared and photopolymerized as previously reported [40,41,42]. Briefly, PEG-bis-AA (6% *w*/*v*), HA-DXM (0.72% *w*/*v*), and Irgacure 2959 (0.1% *w*/*v*) were dissolved in Dulbecco’s phosphate-buffer saline (PBS) without Ca^++^ and Mg^++^. Hydrogel disks were photopolymerized between two coverslips separated by 0.5 mm spacers using low-intensity UV light (365 nm, 10 mW/cm^2^, Black-Ray B100-AP, Upland, CA, USA) for 5 min per side (16 µL of PEG-bis-AA/HA-DXM macromer solution/gel). PEG-bis-AA/HA-DXM hydrogels had a diameter of 5 mm, a thickness of 0.5 mm, and contained approximately 3 μg of DX/hydrogel. Hydrogels were stored in PBS at 4 °C overnight before implanting in moderate-TBI model.

### 5.2. Animal Care and Surgical Procedure

Twenty Male Sprague Dawley rats (8–9 weeks, Charles River, Wilmington, MA, USA) between 290 and 350 g were randomly assigned to one of three groups: (1) Normal group: no surgery, (2) untreated TBI group, and (3) PEG-bis-AA/HA-DXM gel-treated group (3 μg of DX/hydrogel). Rats were housed in 12 h light/dark cycle at Godley-Snell Research Center (GSRC), Clemson University. All animal care, maintenance and experimental procedures were approved by the Clemson University Institutional Animal Care and Use Committee (IACUC).

Generation of the moderate-CCI injury model in rats was performed as described in our previous publication [45]. Briefly, rats were anesthetized using an intraperitoneal (IP) injection of ketamine (80 mg/kg) and xylazine (10 mg/kg). The head was secured in a stereotaxic frame (David Kopf Instruments, Tujunga, CA, USA) and the surgical area was scrubbed with alcohol and betadine. Under aseptic conditions, a midline incision was performed, and the skull was cleared of connective tissue. A 6 mm craniectomy was performed using a trephine burr, over the right hemisphere (midpoint 3.0 mm lateral, 1.0 mm caudal to bregma) without disruption of the dura. A moderate CCI injury was generated using a TBI impactor (Precision Systems and Instrumentation) armed with a 5 mm blunt end tip at set velocity of 4 m/s, depth of 2.5 mm below the surface of the parietal cortex, and dwell time of 250 msec [42]. A polypropylene ring (approximately 7 mm in diameter, 3.5 mm in height) was secured using acrylic resin (Ortho-Jet BCA, Lang Dental Manufacturing Company, Inc., Wheeling, IL, USA) to frame the craniectomy site and maintain gel positioning. Hydrogels were placed on top of the injured cortex. The incision site was sutured using 4-0 vicryl (polyglactin 910) suture, and the rats were recovered.

### 5.3. Cognitive Function Using Morris Water Maze (MWM) Test

**Scheme 1:**
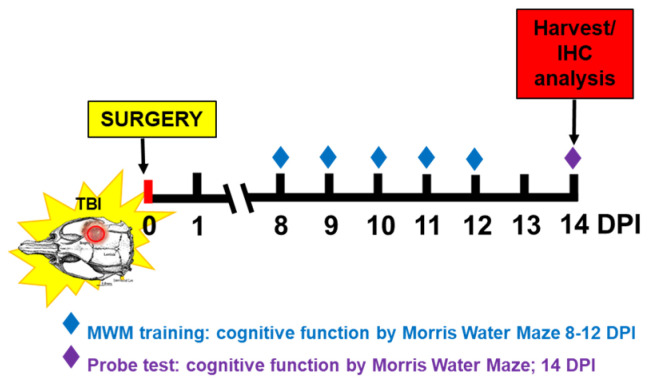
Experimental design and timeline of cognitive function and histological assessment.

MWM test was performed from 8 DPI after the incision had healed. MWM test comprised 4 trials per day over a 5-day training period, with a final “probe test” at 14 DPI (Scheme 1).

A circular pool (183 cm diameter) filled with water was maintained at 25 °C and located in a dimly lit room. Non-toxic black paint, Prang (Dixon Ticonderoga company, Lake Mary, FL, USA) was added to the water to make it dark and opaque. To evaluate spatial learning and memory function, visual cues were placed around the pool within the line of sight of the rats. A circular platform (15 cm diameter) was placed in a fixed location in the pool and submerged 2 cm below the water surface. Training began at 8 DPI and continued for 5 consecutive days (4 acquisition trials/day). Trials were performed according to previously published procedure [41]. During the trials, the swim paths were recorded using a video tracking system (SMART, Panlab, Harvard Apparatus, Holliston, MA, USA) Ho Chi Minh City, Vietnam), and the search time to find the platform was recorded. For the probe test, the platform was removed from the tank, and latency was recorded as the time taken to locate the former platform location and remain there for at least one second. Video recording and analysis were performed using the PanLab SMART 3.0 software [56,57].

### 5.4. Tissue Harvest and Histology Preparation

Brain tissue was harvested at 14 DPI after MWM test. Before sacrifice, rats were deeply sedated with an i.p. injection of EUTHASOL^®^ (150 mg/kg pentobarbital sodium, Virbac, Westlake, TX, USA). A midline thoracotomy was performed, and the rats were transcardially perfused with ice cold 0.9% saline to remove blood followed by cold 4% paraformaldehyde solution (PFA). Brains were isolated and post-fixed in PFA for 24 h at 4 °C. The isolated brains were rinsed using PBS before saturation in increasing concentrations of sucrose (10, 20, and 30% in PBS) and stored in 30% sucrose at 4 °C. For sectioning, brains were rinsed in PBS and embedded in Tissue Plus OCT compound (Fisher HealthCare, Huston, TX, USA). Samples were flash frozen in Freeze-It (Fisher Scientific, Kalamazoo, MI, USA) and stored at −20 °C for two hours. Cryo-sectioning was performed on a Leica CM cryostat (CM1950, Leica Biosystems, Buffalo Grove, IL, USA). Coronal sections were made at 30 µm thickness and stored in cryoprotectant (30% sucrose, 1% polyvinylpyrrolidone, and 30% ethylene glycol in 1X PBS) at −20 °C until ready for histological analysis.

### 5.5. Histological Analysis

#### 5.5.1. Lesion Volume Analysis

Lesion volume was determined by Nissl staining of sequential sections at 0.36 mm intervals within the lesion. Brain sections were washed in three changes of 0.1 M PBS, mounted on charged slides, and allowed to dry overnight. The next day, sections were washed in two changes in deionized, distilled water and then stained with 1% cresyl violet for 5 min. Sections were dehydrated in increasing concentrations of alcohol (50, 70, 90, and 100%), optically cleared in xylene, coverslipped with dibutylphthalate polystyrene xylene (DPX) mounting media (Electron Microscopy Science, Hatfield, PA, USA) and allowed to cure at room temperature. Brightfield images were taken using the 10× objective on a Keyence All-in-One fluorescence microscope (Keyence BZ-X810, Osaka, Japan). Using ImageJ open-source software (1.54i 03), the area of lesion per section was measured, and the total volume of the brain lesion was determined using Cavalieri’s equation of approximation [41].

#### 5.5.2. Assessment of Inflammatory Response and Astrogliosis via Immunohistochemistry

To evaluate the effect of PEG-bis-AA/HA-DXM hydrogel on macrophage infiltration, astrogliosis, and neuron cell survival at 14 DPI, IHC staining was performed as previously described [45]. Briefly, sections were identified at the epicenter and +/−0.72 mm, +/−2.44 mm, +/−2.26 mm and +/−2.98 mm to the epicenter (total 9 sections/rat; Normal n = 3; TBI (untreated) n = 9; TBI + PEG-bis-AA/HA-DXM n = 8). Free floating sections were washed in three changes of 0.1 M PBS before blocking in a solution containing 0.1% Tween 20 (BP337-500, Fisher Chemical, Waltham, MA, USA), 1% BSA (Rockland antibodies and assays, Limerick, PA, USA), and 4% normal goat serum (ab7481, Abcam, Cambridge, MA, USA) for 1 h at room temperature. The sections were incubated overnight at 4 °C in primary antibody solution (PBST with 1% BSA) with gentle rocking. Primary antibodies included Anti-GFAP antibody targeting glial fibrillary acidic protein in astrocytes (ab7260, Abcam, Cambridge, MA, USA), Anti-NeuN antibody, clone A60 targeting neuronal cells (MAB 377, Millipore Sigma, Temecula, CA, USA), Anti-macrophage/monocyte antibody, clone ED1 (MAB1435, Millipore Sigma, Temecula, CA, USA), and ARG1/Arginase 1 Antibody (E-2) (SC-271430, Santa Cruz Biotechnology, Santa Cruz, CA, USA). Double staining was performed with GFAP (1:500 dilution)/NeuN (1:200 dilution) and ED1 (1:200 dilution)/Arg1 (1:250 dilution). The following day, sections were washed in PBST and then incubated for 2 h at room temperature in secondary antibody solution (PBST supplemented with 1% BSA). Secondary antibodies were AF488-conjugated goat anti-rabbit (1:250, A-11008, ThermoFisher Scientific, Rockford, IL, USA) and Cy3-conjugated goat anti-mouse (1:200, 115-165-003, Jackson Immuno Research, West Grove, PA, USA). After incubation, sections were washed with 2 changes in PBST, a final wash with 0.1 M PBS, and then coverslipped with VECTASHIELD Vibrance antifade mounting media with DAPI (H-1800, ThermoFisher Scientific, Eugene, OR, USA). Images of the perilesional cortex were taken using 10× objective on Keyence BX-810 All-in-One fluorescence microscope. Analysis was performed using ImageJ. ED1^+^ and Arg1^+^ cells were identified with the DAPI overlay and normalized to area (mm^2^). NeuN^+^ cells were identified by co-localization with DAPI nuclear staining. Total DAPI^+^ nuclei were quantified to represent the total number of cells, and the results are presented as the percentage of NeuN^+^ cells relative to the total number of DAPI^+^ nuclei. GFAP expression was analyzed as fluorescent intensity and normalized to the corresponding location on an uninjured brain.

#### 5.5.3. Apoptosis by TUNEL Assay

Apoptotic cells were detected using the ApopTag^®^ Plus Fluorescein In Situ Apoptosis detection kit (S7111, Millipore, Burlington, MA, USA) for terminal deoxynucleotidyl transferase dUTP nick-end (TUNEL) labeling at 14 DPI. The sections were again identified at the epicenter, +/− 0.72 mm, +/− 2.44 mm, +/− 2.26 mm, and +/− 2.98 mm to the epicenter (total of 9 sections/rat) and mounted on silane-coated slides (63411-01, Electron Microscopy Science, Hatfield, PA, USA). The sections were washed in 3 changes of 0.1 M PBS. The staining was carried out according to the manufacturer’s specifications using materials provided.

Briefly, sections were post-fixed for 5 min in pre-cooled (−20 °C) ethanol–acetic acid (2:1) solution, then washed with PBST 3 times for 5 min/wash. The sections were treated with the equilibration buffer and incubated with TdT enzyme diluted in primary antibody dilutant (PBST with 1% BSA) solution (7:3 dilution) at 37 °C for 1 h. Following incubation, the sections were submerged in stop/wash buffer for 10 min and washed with PBS. The fluorescein-labeled anti-digoxigenin conjugate was applied and allowed to incubate for 30 min at room temperature protected from light. The slides were washed in PBS and then coverslipped with VECTASHIELD Vibrance antifade mounting media with DAPI. The perilesional cortex was imaged using a Keyence BX-810 fluorescence microscope with the 10× objective. TUNEL^+^ cells were identified by co-localization with DAPI^+^ cells. The number of DAPI^+^ cells were quantified for the total number of cells, and the results are presented as the percentage of TUNEL^+^ cells relative to the total number of cells.

### 5.6. Statistical Analysis

Statistical analysis was performed using one-way ANOVA followed by Tukey’s post hoc test used for pairwise comparison. Data are presented as the mean  ±  SEM (standard error of the mean). Results were considered significant for *p* value < 0.05 on a 2-tailed test.

## Figures and Tables

**Table 1:** Key findings from PEG-bis-AA/HA-DXM hydrogel treatment on functional recovery and secondary injury observed in rat mild and moderate-CCI TBI models at 7 and 14 DPI.

	MILD TBI		Moderate TBI	
Assessment	7DPI	14 DPI	7DPI	14 DPI
Lesion volume	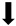	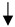	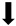	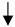
Macrophage/Microglia	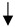	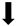	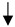	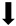
Reactive gliosis (GFAP)	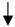	N/A	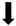	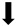
Apoptosis	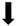	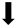	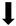	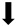
Inflammatory gene expression	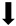	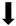	N/A	N/A
Neuronal cell survival	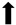	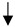	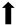	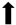
Motor function	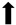	N/A	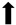	N/A
Cognitive Function	N/A	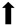	N/A	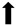

Histological, molecular, and functional outcomes from PEG-bis-AA/HA-DXM hydrogel treatment relative to untreated TBI group at 7 DPI [40] and 14 DPI [41] in a rat mild-TBI model and 7 DPI [42] and 14 DPI in a rat moderate-TBI model. Changes in each outcome assessment within each individual study are indicated as statistically significantly increase (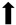) and significantly decrease (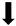), qualitative or not statistically significantly decrease (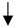), and not assessed (N/A).

## Data Availability

Data will be made available on request.

## Abbreviations

The following abbreviations are used in this manuscript:

TBI	Traumatic Brain Injury
DX	Dexamethasone
PEG-bis-AA	Polyethylene glycol-bis-(acryloyloxy acetate)
HA-DXM	Dexamethasone-conjugated hyaluronic acid
CCI	Controlled cortical impact
BBB	Blood–brain barrier
GFAP	Glial fibrillar acidic protein
NeuN	Neuronal nuclei
DPI	Days post-injury
MWM	Morris Water Maze
IHC	Immunohistochemistry
PBS	Phosphate-buffered saline
ECM	Extracellular matrix
IACUC	Institutional Animal Care and Use Committee
IP	Intraperitoneal
PFA	Paraformaldehyde
DI H_2_O	Deionized H_2_O
DPX	Dibutylphthalate polystyrene xylene
BSA	Bovine serum albumin
TUNEL	Terminal deoxynucleotidyl transferase dUTP nick-end
DAPI	4′,6-diamidino-2-phenylindole
